# A Bioanalytical Platform for Simultaneous Detection and Quantification of Biological Toxins

**DOI:** 10.3390/s120202324

**Published:** 2012-02-21

**Authors:** Oliver G. Weingart, Hui Gao, François Crevoisier, Friedrich Heitger, Marc-André Avondet, Hans Sigrist

**Affiliations:** 1 Laboratory of Food Microbiology, Institute of Food, Nutrition and Health (IFNH), ETH Zurich, Schmelzbergstrasse 7, 8092 Zurich, Switzerland; 2 Toxinology Group, SPIEZ LABORATORY, 3700 Spiez, Switzerland; E-Mail: marc.avondet@babs.admin.ch; 3 Arrayon Biotechnology SA, Jaquet-Droz 1, 2002 Neuchâtel, Switzerland; E-Mails: hui.gao@arrayon.com (H.G.); francois.crevoisier@arrayon.com (F.C.); friedrich.heitger@arrayon.com (F.H.); hans.sigrist@arrayon.com (H.S.)

**Keywords:** biosensor, microfluidics, toxins, bioanalytics, platform

## Abstract

Prevalent incidents support the notion that toxins, produced by bacteria, fungi, plants or animals are increasingly responsible for food poisoning or intoxication. Owing to their high toxicity some toxins are also regarded as potential biological warfare agents. Accordingly, control, detection and neutralization of toxic substances are a considerable economic burden to food safety, health care and military biodefense. The present contribution describes a new versatile instrument and related procedures for array-based simultaneous detection of bacterial and plant toxins using a bioanalytical platform which combines the specificity of covalently immobilized capture probes with a dedicated instrumentation and immuno-based microarray analytics. The bioanalytical platform consists of a microstructured polymer slide serving both as support of printed arrays and as incubation chamber. The platform further includes an easy-to-operate instrument for simultaneous slide processing at selectable assay temperature. Cy5 coupled streptavidin is used as unifying fluorescent tracer. Fluorescence image analysis and signal quantitation allow determination of the toxin’s identity and concentration. The system’s performance has been investigated by immunological detection of Botulinum Neurotoxin type A (BoNT/A), Staphylococcal enterotoxin B (SEB), and the plant toxin ricin. Toxins were detectable at levels as low as 0.5–1 ng·mL^−1^ in buffer or in raw milk.

## 1. Introduction

Despite intensified efforts, intoxication by toxins such as the bacterial toxins Staphylococcal enterotoxin B (SEB) or Botulinum Neurotoxin type A (BoNT/A) still present significant threats to public health [1]. Ingestion of SEB, produced by gram positive cocci of *Staphylococcus aureus*, can cause Staphylococcal Food Poisoning (SFP), a form of gastroenteritis that manifests clinically as emesis with or without diarrhea. As little as 1–20 μg SEB may lead to severe illness in an adult [2,3]. BoNT/A, /B, /E, and /F, are produced by toxigenic strains of the Gram positive *Clostridium botulinum* and may provoke food borne botulism in humans, a severe neuroparalytic illness. BoNT is the most toxic substance known to man. Quantities of 0.01–1 μg BoNT per kg body weight can be fatal if ingested, e.g., with improperly processed food [4,5].

With more than 200,000 estimated intoxication cases in USA in 2006, SEB caused costs of approximately $1.2–1.5 billion for appropriate treatment, thus generating an enormous economic loss. Between 1992 and 1997 there occurred only about 60 cases of food borne botulism in the USA per year, with often very severe etiopathology. Treatment in intensive care units is often inevitable, and in certain cases artificial ventilation during up to 6 months is required. Such medical treatment increases the costs per patient to $14,000–75,000 and may reach annual care costs of $800,000 to $4.2 million [6–9]. Another dreaded toxin is ricin, a toxic lectin of the castor oil plant *Ricinus communis*. While seeds of *R. communis* are pressed cold to extract the oil, water-soluble ricin remains after processing within fibrous residues. Ricin is a prototypic A–B toxin, which effectively inhibits protein synthesis by depurinating the 28S ribosomal RNA. The actual oral toxicity of ricin for humans is estimated 1 to 30 mg per kg body weight. Nevertheless, facile extraction and industrial castor oil production lead to toxin abundance and facilitated access [10,11]. Hence, ricin is regarded as threatening biowarfare agent. Likewise, due to their exceptional toxicity, both SEB and BoNT are considered potential biowarfare agents [12]. It is thus considered appropriate to develop tools and procedures applicable for fast and early detection and typing of toxins for food safety and homeland security.

Over the last five decades, the development of biosensors has attracted considerable interest, and the number of analytes detected increases continuously [13–15]. Though most biosensors are currently used in clinical diagnosis [16], they also gain importance for applications such as DNA hybridization and sequencing [17], high throughput drug discovery [18,19], detection of biological warfare agents [20], environmental analysis, e.g., of pesticides [21], food production and safety [22], and detection of illicit drugs [23,24]. Microfluidic biosensing systems in particular have gained attention, as they offer advantages over other biosensors. Commonly, they include fluid channel systems imprinted in plastic or silicone platforms [25,26]. In many instances, channels are connected to appropriate fluid propagation systems allowing fluid transport to and from reaction chambers. Depending on the microfluidic channel arrangement, selective contact of analyte-containing sample fluids with distinct areas of the analytical platform is assured [27]. Minute sample volumes are generally needed due to the nanometer to micrometer channel dimensions. This renders large amounts of expensive reagents superfluous, and assays can be performed with scarce and precious samples.

Currently, an increasing number of Lab-on-a-chip devices are emerging. Due to their ability to test multiple samples with small amount of reagents, they offer optimal conditions to detect highly contagious or harmful substances. While a wide range of methods is used to address this matter, many assays rely on immunological detection of target molecules due to the high specificity and sensitivity of immunological reagents. This is of particular importance when considering the high toxicity of certain toxins, foremost BoNT/A, where detection in the range of few picogram per milliliter is required. To guarantee food safety, at least 125–250 ng of SEB per 100 g of a given food sample should be detected [28]. This corresponds to approximately 1.25–2.5 ng·mL^−1^ liquid foodstuff. Although the toxicity of ricin is much lower, it is evident that safety margins are required, *i.e.*, a detection limit of few nanogram per mL would be eligible. Accordingly, there is need for sensitive and reliable detection of such toxins.

If proteinaceous or peptidic toxins are to be detected, immunological detection techniques, such as enzyme-linked immunosorbent assay (ELISA) or fluorescence-linked immunosorbent assay (FLISA), are currently the methods of choice, as they offer both high sensitivity and specificity [29,30]. Accordingly, many biosensors utilize capture molecules such as antibodies immobilized in discrete locations on the surface of microfluidic platforms [27,31–33]. By passing the detection area, analytes may interact with capture molecules. Captured analytes can subsequently be detected immunologically, by hybridization or by direct molecular detection. An often-employed sensing scheme is based on fluorescence detection: a fluorescent label coupled to a detection molecule serves to identify and quantify captured analytes [34,35]. Sandwich assays employing two specific antibodies, allow highly sensitive and specific analyte detection—even in complex matrices [36]. Immobilization of different capturing molecules to analytical platforms enables both, simultaneous detection of several analytes and integration of relevant positive and negative control features [37].

The present contribution describes a bioanalytical platform, which combines the specificity of covalently immobilized capture probes with a dedicated instrumentation and microfluidic array analytics. A set of toxin specific antibody pairs is used to challenge the portrayed bioanalytical system with three biological threat agents BoNT/A, SEB, and ricin. The described system allowed for simultaneous characterization of toxin type and concentration. While optimum conditions for highest sensitivity were established in buffer, the system also qualified for the detection of BoNT/A and ricin in a complex matrix such as raw milk.

## 2. Experimental Section

### 2.1. Toxins and Antibodies

The study was performed using purified 150 kDa BoNT/A from *Clostridium botulinum* strain Hall A (BoNT/A1; Metabiologics, Madison, WI, USA), ricin from *Ricinus communis* (RCA60; Institute of Phytochemistry, University Witten/Herdecke, Witten, Germany), and SEB from *Staphylococcus aureus* (S4881; Sigma-Aldrich, Seelze, Germany). For sample analysis, toxins were diluted in pH 7.2 phosphate buffered saline (PBS), pH 7.4 Tris buffered saline (TBS) or raw cow’s milk (pH 6.5, approximately 4% fat content), respectively. The following mouse-derived monoclonal antibodies were used as capturing antibodies: anti-BoNT/A (A1688; ZBS 3, RKI, Berlin, Germany) as described before [38], anti-SEB (89/3; Institute for Medical Microbiology and Hygiene, TU Dresden, Dresden, Germany), and anti-Ricin (RCH1; Institute of Phytochemistry, University Witten/Herdecke, Witten, Germany). Detection of the toxins was performed with polyclonal equine anti-BoNT/A (BoNT/A/B/E; Novartis Behring, Marburg, Germany), polyclonal rabbit anti-SEB (S9008; Sigma-Aldrich, Seelze, Germany), and monoclonal mouse anti-ricin (1RK1; Institute of Phytochemistry, University Witten/Herdecke, Witten, Germany). The detection antibodies were coupled to biotin according to the manufacturer’s instructions (EZ-Link Sulfo-NHS-LC-biotin; Pierce, Rockford, IL, USA). They were used at concentrations between 5–10 μg·mL^−1^. Biotinylated antibodies were stored in phosphate buffered saline with 0.2% (w/v) bovine serum albumin and 0.05% (w/v) NaN_3_.

### 2.2. Inca Bioanalytical System

**Figure 1.**
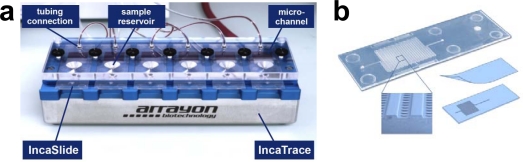
Inca Bioanalytical System. (**a**) The IncaTrace instrument is equipped with an integrated heating plate. Six IncaSlides are mounted on the IncaTrace instrument and connected via Tygon ST tubings to a multi-channel peristaltic pump; (**b**) IncaSlide with microstructured channel architecture and lamination. The inset shows a section (SEM image) of substructured microchannels. The diameter of the protruding posts is 300 μm.

The Inca Bioanalytical System is a microarray-based system for use in diagnostics and environmental control. The current configuration of the system consists of two essential components: IncaTrace (Figure 1(a)) and IncaSlide (Figure 1(b)), the first being a small and easy-to-use laboratory appliance for simultaneous IncaSlide processing. Toxin assays were performed with the Inca Bioanalytical System depicted in Figure 1.

### 2.3. IncaSlide

**Figure 2.**
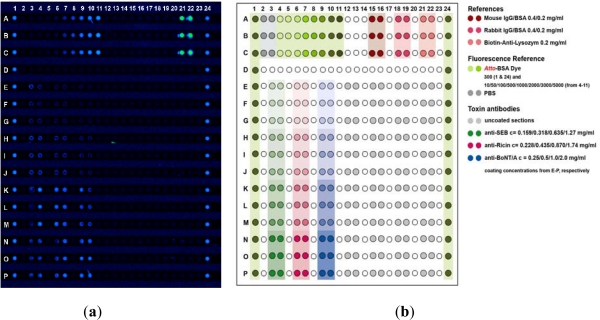
Print layout of an IncaSlide. (**a**) Fluorescence scan image of a processed IncaSlide. The size of the analyzed area is 16 × 12 mm (≈2 cm^2^). Reference sidelines serve to control print and slide quality. In the example shown, a fluorescence intensity calibrator series is deposited at the top of the microarray. Baseline values are retrieved from capture-free vertical and perpendicular spot arrays. The slide, functionalized with capture antibodies, was incubated with SEB, Ricin, and BoNT/A at concentrations of 50 ng·mL^−1^ each; (**b**) Layout scheme of the presented slide. Colored areas indicate sections functionalized with capture antibodies (anti-SEB, anti-Ricin, anti-BoNT/A), negative controls (PBS), positive controls (Biotin-anti-Lysozyme), antibody controls (mouse IgG/BSA, rabbit IgG/BSA) and fluorescent dye references (Atto-BSA dye in different concentrations). Increased color intensity represents increased antibody or dye concentrations.

Contrary to most commercialized high performance microarray platforms, the IncaSlide is manufactured by injection molding. The slide’s dimensions fully comply with international standards (25 × 75.7 × 1 mm). The basic architecture of the IncaSlide includes a substructured microchannel with a channel width of 500 μm. Molecules are printed onto 384 protruding posts situated within the channel (Figure 1(b)). A low volume reaction chamber (28 μL) is generated by lamination of the IncaSlide. Prior to dispensing the capture antibodies, the entire channel is treated with the photolinker polymer OptoDex™, and capture molecules are printed using a non-contact microdispensing array printer (Nano-Plotter™, GeSIM, Germany). For microarray manufacturing, capture antibodies were diluted with 1% (w/v) PBS buffer at concentrations ranging from 0.1–2.0 mg·mL^−1^ and dispensed on top of the substructure features (400 pL per feature). Printed arrays were then vacuum dried and slides were exposed to UV light (350 nm, 11.4 mW per cm^2^) for 4 min to achieve covalent binding of the capture molecules. Following photo-immobilization, the slides were rinsed three times each with PBS buffer containing 0.05% Tween 20, PBS buffer and deionized water, respectively, and blocked with Inca Block 6000 (Arrayon Biotechnology, Neuchatel, Switzerland). Upon drying, printed slides were laminated with a transparent acrylate film, packaged under oxygen-free atmosphere (400 mbar N_2_) and stored at 4 °C. All microarray manufacturing procedures were carried out in clean room environment. Besides capture molecules, the microarray is designed to host analytical references and calibrators as required for quality controlled, unambiguous and quantitative analysis of analyte fluids. Packaged microarrays can be stored at ambient temperature. Storage at 4 °C, however, is recommended. Figure 2 shows a typical fluorescence scan image of a processed IncaSlide as well as the underlying layout scheme.

### 2.4. IncaTrace

IncaTrace is small laboratory instrument designed for processing IncaSlides. The instrument consists of a solid base plate with six recesses, a cover plate with fluid connections and fluid reservoirs and screws to tighten the properly positioned IncaSlides. Inlet and outlet ports of the IncaSlide connect to the reservoir (at inlet) and the tubing connection (at outlet), respectively. For processing, up to six IncaSlides are placed in the respective recesses, with the laminated surface down. The cover plate is overlaid and tightened by screws. Buffer solutions, analytes and reagents (max. 100 μL) are sequentially manually dispensed into the fluid reservoirs. Fluids are channeled in the reaction chamber by a multiplex peristaltic pump (IPC-N8 Multichannel pump, Ismatec SA, Switzerland) with a flux rate of 70 μL·min^−1^. Cyclic reversed flux incubation (20 s for each direction) is performed during two times 16 min at ambient temperature or at 37 °C. Upon completion of the incubation and rinsing steps, IncaSlides are removed from the instrument, delaminated and blown dry with N_2_ before fluorescence scanning.

### 2.5. Fluorescence Scanning and Signal Quantification

Cy5-labeled Streptavidin (5 μg·mL^−1^; Milan Analytica AG, Rheinfelden, Switzerland) was used for fluorescence detection of toxin-antibody complexes. Upon scanning of processed IncaSlides, fluorescence signal intensities were registered and quantified with a commercial instrument and software (GenePix Personal 4100 A; Molecular Devices, Inc., Sunnyvale CA, USA). The relative fluorescence intensity (RFU) of each individual array feature was quantitated by relating measured fluorescence intensities to on-chip fluorescence calibrator intensities. Atto-BSA dye (Arrayon Biotechnology, Neuchatel, Switzerland) was used to calibrate fluorescence intensity. Calibrator features were individually printed in triplicates; they contained 5–5,000 atto dye molecules per μm^2^ at constant protein (BSA) concentration.

### 2.6. Decontamination

Plastic parts of the IncaTrace instrument were regularly decontaminated by incubation in 6% NaOH (30 min) followed by 1% detergent (10 min) and subsequently rinsed with deionized H_2_O (3 times 5 min). Similarly, tubings were flushed with 6% NaOH (10 min), followed by deionized H_2_O (10 min), then 100% ethanol (10 min) and finally rinsed with deionized H_2_O (30 min). IncaSlides are designed for single use. They were discarded after decontamination.

## 3. Results

### 3.1. IncaSlide Processing

IncaSlides included a microstructured channel, which—upon lamination—established a low volume reaction chamber. In a typical analysis, IncaSlides printed with assay specific capture probes, calibrator and control items were placed in the IncaTrace instrument. A multichannel peristaltic pump was connected via fluidic connections. Initializing channel charging with buffer solution (by applying negative pressure) reconstituted the previously dry-immobilized capture molecules. Different dilutions of anti-toxin antibodies were used to determine the optimal print concentration of the capture antibody. As a result, the highest antibody concentration (2.0 mg·mL^−1^ anti-BoNT/A, 1.74 mg·mL^−1^ anti-ricin, and 1.27 mg·mL^−1^ anti-SEB) was chosen as it provided high sensitivity and a good signal to noise ratio.

Assay procedures were initiated by applying the analyte samples in two steps (100 μL each). The first sample charge (100 μL) was placed in the IncaTrace sample reservoir. The efficiency of analyte binding to capture molecules was enhanced by cyclic reverse flow incubation for 16 min. After discharging the channels, the second sample charge (100 μL) was applied and handled the same way as above. Subsequently, 100 μL of biotin labeled detection antibodies was added. For fluorescence detection, fluorophore (Cy5) labeled Streptavidin was used to visualize established complexes of capture antibody, analyte and biotinylated second antibody. Each incubation step took 16 min and was followed by three rinsing steps with PBS buffer containing 0.05% Tween 20, then PBS buffer and deionized water. Processed slides were scanned and the fluorescent signals were quantified. Relating the recorded fluorescence to the intensities of the on-chip fluorescence calibrator allowed quantitation of each individual array feature.

### 3.2. Assay Development

#### 3.2.1. Dose Response *versus* Assay Temperature

**Figure 3.**
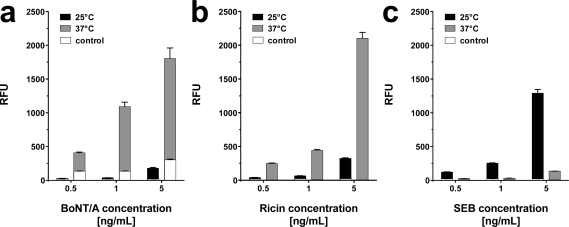
Temperature dependent detection of BoNT/A, ricin, and SEB. PBS was spiked with different concentrations (0.5, 1, and 5 ng·mL^−1^) of (**a**) BoNT/A, (**b**) Ricin, and (**c**) SEB, respectively, and incubated either at 25 °C (black bars) or at 37 °C (gray bars). Negative control values (white) are integrated in the depicted bars. Average signal intensity and the standard deviation (error bars) were calculated from six features (n = 6) per assay.

To elucidate optimal assay conditions, the dose response of the system was determined at 25 °C and 37 °C with toxins diluted in phosphate buffered saline (PBS; pH 7.2). For each toxin concentration an individual slide was used. As depicted in Figure 3, all toxins could be reliably detected at 25 °C at concentrations as low as 0.5 ng·mL^−1^ for SEB and 0.5–1 ng·mL^−1^ for BoNT/A and ricin, respectively. Upon incubation at 37 °C, the signal intensities of BoNT/A and ricin increased significantly, allowing toxin detection at 0.5 ng·mL^−1^. SEB related signals, however were negatively affected by elevated assay temperatures.

#### 3.2.2. Dose Response *versus* Assay Buffer Composition

**Figure 4.**
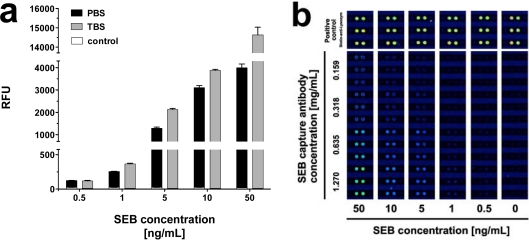
Detection of SEB diluted in PBS and TBS and image of IncaSlide. (**a**) PBS (black bars) and TBS (gray bars) were spiked with SEB (final concentrations 0.5, 1, 5, 10, and 50 ng·mL^−1^) and slides were processed at 25 °C. Negative control values (white) are integrated in the bars. Average total signal intensity and standard deviation (error bars) were calculated from six features (n = 6) per assay; (**b**) Fluorescence scan image series of IncaSlide sections functionalized with anti-SEB capture antibody and SEB applied at concentrations of 0–50 ng·mL^−1^, diluted in TBS. The respective positive controls (Biotin-anti-Lysozyme) are shown in the top part of the image.

In an attempt to find the optimal buffer composition for the simultaneous detection of the toxins, we performed assays with PBS (pH 7.2) and Tris buffered saline (TBS; pH 7.4) at 25 °C. While SEB in PBS displayed very good signal intensities, we found even higher signals with TBS as an assay buffer (Figure 4). For 50 ng·mL^−1^, for example, the signal intensity increased by 72.7%, while the effect was less pronounced at lower concentrations. Signal intensities for 0.5 ng·mL^−1^ SEB, for instance, increased merely by 6.5%. In contrast to SEB, the signal intensities for BoNT/A and ricin decreased dramatically with toxins dissolved in TBS. As a result, solutions of ricin in TBS could only be detected at concentrations ≥ 5 ng·mL^−1^, and concentrations ≥ 50 ng·mL^−1^ were required for detection of BoNT/A in TBS (data not shown).

#### 3.2.3. Dose Response *versus* Incubation Time

In view of further optimizing the assay conditions, extension of the analyte incubation time was explored (twice instead of once 16 min for each sample charge). Results obtained indicated that doubling the incubation time did not increase the signal intensity.

### 3.3. Simultaneous Detection of Toxins

**Figure 5.**
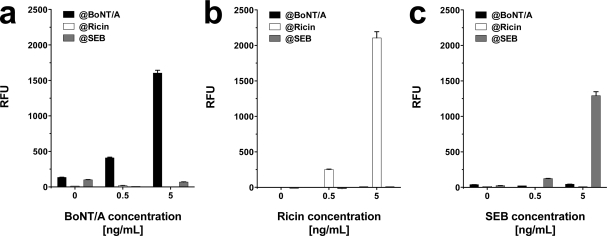
Cross-reactivity of anti-toxin antibodies. PBS was spiked with 0.5 and 5 ng·mL^−1^ of (**a**) BoNT/A, (**b**) ricin, and (**c**) SEB, respectively, and slides were processed at 37 °C (BoNT/A and ricin) and 25 °C (SEB). Signals are shown for the spiked toxin, as well as signals measured at features functionalized with capture antibodies against the other toxins. Signals measured at anti-BoNT/A (@BoNT/A) coated features are depicted in black, signals at anti-ricin (@Ricin) coated features in white, and signals at anti-SEB (@SEB) coated features in gray. Average signal intensity and standard deviation (error bars) were derived from six features (n = 6) per assay.

In addition to demonstrating the detection of toxins in parallel, we tested the system’s performance by detecting different toxins simultaneously on one slide. Prior to these investigations the cross-reactivity of the antibody pairings was determined. Toxins were diluted in PBS at concentrations of 0.5 and 5 ng·mL^−1^ and the slides were incubated with one toxin at the time at a concentration of either 0.5 or 5 ng·mL^−1^. Next, a mixture of biotin labeled secondary antibodies specific for all three toxins, and subsequently Cy5 labeled streptavidin were applied. The fluorescence intensities at features functionalized with capture antibodies, which do interact with the toxin in question were compared to the respective negative control without toxin. As shown in Figure 5, even at a tenfold increase of spiked toxin the amount of unspecific signal, registered on the features specific for non-target toxins, did not increase significantly. The results imply minimal cross-reactivity between capture and detection antibodies, as well as between toxins and non-pairing capture antibodies.

**Figure 6.**
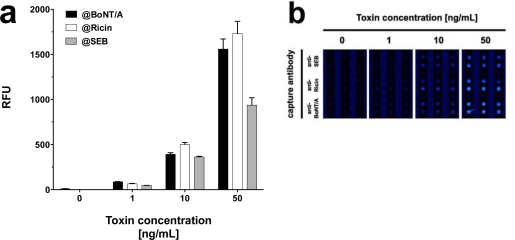
Simultaneous detection of BoNT/A, ricin and SEB in PBS. PBS was spiked with equal concentrations (0, 1, 10, and 50 ng·mL^−1^) of all three toxins and slides were processed at 37 °C. (**a**) Values are shown for the signals measured at features functionalized with the respective capture antibodies, *i.e.*, anti-BoNT/A (@BoNTA; black bars), anti-ricin (@Ricin; white bars), and anti-SEB (@SEB; gray bars). Average signal intensities and the standard deviations (error bars) were calculated from six measurements (n = 6) per assay; (**b**) Image series of features functionalized with the highest concentrations for anti-SEB, anti-ricin, and anti-BoNT/A, respectively, and challenged with a mixture of either 0, 1, 10, or 50 ng·mL^−1^ of the three toxins.

As all three toxins showed significant signals when assayed in PBS, this buffer was chosen for simultaneous toxin detection. To attain this, PBS was spiked with equal concentrations of all three toxins, *i.e.*, 1, 10 or 50 ng·mL^−1^. Similar to singleplex assays, each mixture of equally concentrated toxins was incubated on a single slide at 37 °C. Although generally lower signals were observed, the signal intensities were sufficiently high to detect all three toxins simultaneously at concentrations as low as 1 ng·mL^−1^ (Figure 6). Interestingly, in spite of incubation at 37 °C, signal intensities for SEB did not decrease notably and quantification was possible, even with mixtures of toxins at different concentrations (data not shown).

### 3.4. Detection of Toxins in a Complex Matrix

It has been discussed in the relevant literature [39], whether BoNT/A might be used in case of a bioterror attack to contaminate the food supply, *i.e.*, the milk production. Accordingly, it seemed pivotal to test the bioanalytical system with toxins in raw milk as food matrix. Due to its high protein and lipid content, raw milk represents a complex and thus challenging matrix. In the context of the present investigations, the toxins were diluted in raw milk and applied to the bioanalytical platform without prior sample preparation.

**Figure 7.**
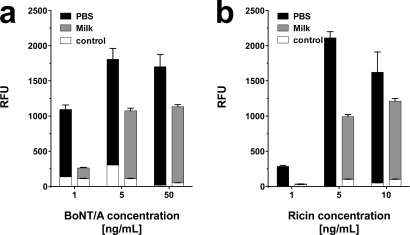
Dose response for BoNT/A and ricin in raw milk. PBS (black bars) and raw milk (gray bars) were spiked with different concentrations of (**a**) BoNT/A (1, 5, and 50 ng·mL^−1^) and (**b**) ricin (1, 5, and 10 ng·mL^−1^). Negative control values are depicted in white within the respective measurement. Average signal intensities and the standard deviation (error bars) were calculated from six features (n = 6) per assay.

As shown in Figure 7, BoNT/A and ricin were detected in raw milk at concentrations as low as 1 and 5 ng·mL^−1^, respectively. Unexpectedly, detection of SEB in raw milk was only possible at concentrations ≥ 50 ng·mL^−1^ (data not shown). A minor increase in background signal was observed for ricin. False positives or false negatives were not detected. Hence, although raw milk as a complex matrix impaired the assay, in particular for SEB, it was still possible to detect low concentrations of BoNT/A and ricin.

## 4. Discussion and Conclusions

The primary goal of this study was to demonstrate validity of design and performance of a scalable, microfluidic device for parallel and simultaneous detection of specific antigens. Therefore, an assay was developed consisting of capture antibody-functionalized slides with microfluidic channels connected to an appropriate instrument with sample reservoir and connection to a fluid propagation system. Capture antibodies were photochemically immobilized in the dry state using OptoDex™, a dextran based linker polymer, to obtain covalent capture binding on top of sub-microstructured channels [40,41]. The performance of the bioanalytical system was challenged with three toxins, which present a major threat to food industry and public health. BoNT/A, Ricin, and SEB were examined, and the assay parameters: buffer composition, incubation temperature, incubation time, assay format (single or simultaneous) as well as matrix effects were investigated.

The presented data show that the Inca Bioanalytical System enables the detection of 0.5 ng·mL^−1^ of BoNT/A, ricin, or SEB in buffer. An increase in sensitivity was observed for BoNT/A and ricin when the incubation temperature was shifted from 25 °C to 37 °C. While detection of SEB was negatively affected by elevated temperature, the use of TBS as sample buffer exhibited a positive effect on the binding affinity between SEB and its antibodies. Although the exact mechanism is still largely unknown, it has been reported before that different buffer compositions may cause protein stabilization or destabilization [42–44]. Hence, we believe that binding properties between SEB and its antibodies were affected in a similar way. Yet, this finding warrants future research. As the antibody combinations used in the assay displayed only minute cross-reactivity, the system qualified for simultaneous detection of the three toxins. Although the presence of all toxins leads to a decrease in signal intensities, toxin concentrations as low as 1 ng·mL^−1^ were detectable. If raw milk was used as sample matrix, the procedure allowed detection of 1 and 5 ng·mL^−1^ of BoNT/A and ricin, respectively. Accordingly, raw milk partially impaired the sensitivity for these toxins. For SEB however, incubation with raw milk at 37 °C leads to a dramatic decrease in signal intensities. In addition, unspecific binding of milk components to the anti-SEB capture antibody or to the toxin itself may have interfered.

As far as sensitivity is concerned, the presented data correspond to the detection limits of similar biosensor devices for the detection of the investigated toxins. Other publications describe sensitivities in buffers and some food samples as low as 1–20 ng·mL^−1^ for BoNT/A, 10 ng·mL^−1^ for ricin, and 0.1–4 ng·mL^−1^ for SEB, using different forms of flow-coupled immuno-analysis [45–48]. Introduction of enrichment steps, e.g., by antibody-coupled paramagnetic beads may further increase the sensitivity to few picogram per milliliter [49–51]. Using planar waveguide sensors, sensitivities for BoNT/A toxin or toxoid, ricin, and SEB were reported in the range of few ng·mL^−1^ in buffer and milk, respectively [27,33]. Methods, such as capillary electrophoresis combined with laser-induced fluorescence detection, mass-sensitive magneto elastic sensors or diffractive grating sensors reportedly detect SEB, ricin, or BoNT/A at concentrations between 0.5 and 100 ng·mL^−1^ [52–54].

The key strength of the presented bioanalytical platform is its ability to analyze, on a modular scheme, multiple samples for multiple analytes simultaneously with multiple reaction media. The instrument allows fast assay development and exploration of different assay strategies, *i.e.*, buffer compositions, varying sample volumes, flow specifications, incubation times and pre-defined temperature settings. As the entire process can be completed in less than 90 min it is faster than common ELISA procedures and overcomes the sensitivity limitations of existing lateral flow devices [55–57]. In addition, if further detection molecules were incorporated, the system would facilitate simultaneous detection of up to 28 agents with six replicates each on the same slide. Due to inexpensive production, IncaSlides reduce assay costs. Also, they can be easily disposed of, thereby minimizing the risk of post-assay contamination or intoxication, respectively. All parts of the IncaTrace are amenable to decontamination strategies. Hence, by applying the appropriate assay conditions and specific detection molecules, the bioanalytical platform qualifies for sensitive detection of a wide range of molecules, including toxins, simultaneously and in parallel.
